# An Approach of Vibration Compensation for Atomic Gravimeter under Complex Vibration Environment

**DOI:** 10.3390/s23073535

**Published:** 2023-03-28

**Authors:** Hao Che, An Li, Zhu Zhou, Wenbin Gong, Jinxiu Ma, Fangjun Qin

**Affiliations:** School of Electrical Engineering, Naval University of Engineering, No.717 Jiefang Road, Wuhan 430033, China

**Keywords:** atomic gravimeter, vibration compensation, transfer function, equilibrium optimizer algorithm

## Abstract

Atomic gravimeter has been more frequently applied under complex and dynamic environments, but its measurement accuracy is seriously hampered by vibration-induced noise. In this case, vibration compensation provides a way to enhance the accuracy of gravity measurements by correcting the phase noise that resulted from the vibration of a Raman reflector, and improving the fitting of an interference fringe. An accurate estimation of the transfer function of vibration between the Raman reflector and the sensor plays a significant role in optimizing the effect of vibration compensation. For this reason, a vibration compensation approach was explored based on EO (equilibrium optimizer) for estimating the transfer function simplified model of a Raman reflector, and it was used to correct the interference fringe of an atomic gravimeter. The test results revealed that this approach greatly restored the actual vibration of the Raman reflector in a complex vibration environment. With a vibration compensation algorithm, it achieved the correction and fitting of the original interference fringe. In general, it dramatically reduced the RMSE (root mean square error) at the time of fitting and significantly improved the residual error in the gravity measurement. Compared with other conventional algorithms, such as GA (genetic algorithm) and PSO (particle swarm optimization), this approach realized a faster convergence and better optimization, so as to ensure more accurate gravity measurements. The study of this vibration compensation approach could provide a reference for the application of an atomic gravimeter in a wider and more complex environment.

## 1. Introduction

Gravity is a resultant force of gravitation and inertial centrifugal force. Absolute gravity measurement has been widely practiced in various fields such as basic physical research, inertial navigation, underwater exploration, mining, resource exploration, environmental monitoring, marine development, earthquake prediction, and global gravity field mapping [1,2,3,4]. Presently, most high-precision absolute gravimeters rely on the free-fall method for an absolute gravity measurement. For instance, a laser interferometry absolute gravimeter (hereinafter referred to as an optical gravimeter) uses a cube-corner prism as the falling object [5,6], and an atomic interferometry absolute gravimeter (hereinafter referred to as an atomic gravimeter) takes a cold atomic group as the falling object [7,8]. The gravimeters are both employed to measure the acceleration of the falling object or atomic group against an internal reference mirror (that is, a cube-corner prism or a plane mirror). In the principle of equivalence, it is impossible for a gravimeter to identify the gravitational acceleration from the acceleration of vibration-induced noise resulting from the external environment. Consequently, the reference mirror is highly sensitive to the external vibration-induced noise. The vibration-induced noise will inevitably exert an effect on the measurement, leading to the measurement error of the gravimeter. As revealed in some test results, an accurate gravity measurement is not easily implemented if the vibration-induced noise is not inhibited [9].

Vertical vibration isolation and vibration compensation are two approaches predominantly adopted to cope with the vibration-induced noise in the absolute gravity measurement. Ultralow frequency vertical vibration isolation is a technology that has existed for a long time. Till today, it has been able to isolate the vibration up to µGal, and satisfy the need for a high-precision absolute gravity measurement. Nevertheless, a vertical vibration isolation system is often used for the absolute gravity measurement on the static ground in a quiet environment such as laboratories because of its poor anti-interference. For instance, a test team of the Laboratoire National de Metrologie et d’Essais-Systemes de Reference Temps-Espace (LNE-SYRTE) [10], a research team from the Stanford University [11], Feier from the Humboldt–Universitat zu Berlin [12], Zhou Minkang from the Huazhong University of Science [13,14] and Technology, and Tang Biao from the Chinese Academy of Sciences [15]. At the end of the 20th century, vibration compensation was put forth to detect the vibration-induced noise of a reference mirror inside a gravimeter using a sensor, such as a seismometer or an accelerometer, and then correct the measured gravity accordingly. The vibration compensation with a seismometer in a laboratory had been achieved by some research teams from the Observatoire de Paris [16], the Universität Hannover [17], the National Institute of Metrology, China [18], the Tsinghua University [19], and the Zhejiang University [9]. After vibration compensation, the standard deviation of each measurement reached dozens or hundreds of µGal. Moreover, the compensation vibration with an accelerometer outdoors or on a mobile platform had been practiced by some research institutes such as the Swiss Federal Institute of Technology Zurich [20], the ONERA [21,22,23], the Zhejiang University of Technology [24], and the Huazhong University of Science and Technology [25]. After compensation vibration, the standard deviation of each measurement was up to mGal. Evidently, the existing vibration compensation systems can achieve accuracy up to mGal to dozens of µGal, which is inferior to a vertical vibration isolation system. However, a vibration compensation system features a simple structure and offers greater anti-interference. If a measurement is carried out outside a laboratory or in a field, that is, a dynamic environment, instead of a static environment, the supporting platform of the gravimeter will be changed and will even start to move. The measurement environment will deteriorate in various aspects, e.g., an increased amplitude of vibration-induced noise and a widened platform. In this case, vertical vibration isolation must be changed to vibration compensation since the latter has approximately the same measurement accuracy but a better performance in a dynamic environment.

Nevertheless, these studies focus on a direct compensation and they overlook the influence of the transfer function between a sensor and a reference mirror. Richardson [17] pointed out that the mechanical transfer function between a reference mirror and a traditional vibration sensor originates from the installation location and mechanical structure. Prior to measurement, it is therefore necessary to initially define the transfer function for better vibration compensation. In 1997, Canuteson et al. [26] employed an absolute gravimeter to define the transfer function of an STS-2 seismometer, and they found it possible to correct the ground noise with a seismometer. Nevertheless, the transfer function of a seismometer may be easily altered by the changing environment or the drift of an instrument, which leads to complex compensation and unsatisfying results. In 2001, Brown et al. [27] put forward a vibration compensation approach in which the information from an external sensor was used for compensation to gather the data of absolute gravity. In the approach, the vibration isolation system of an optical gravimeter FG5 was replaced by a vibration compensation system. The results of static measurement in a laboratory environment showed that this vibration compensation system enhanced the measurement accuracy by around seven times, from ±500 μGal to ±65 μGal; its effect was quite satisfying. In 2017, Wang et al. [18] carried out a theoretical analysis on how the output signal of a gravimeter sensor related to the motion of a reference mirror and estimated the transfer function for such a relation. Furthermore, the transfer function was simplified into a “gain-delay” model after considering the flat response of such a transfer function within the bandwidth of the seismometer, the arrangement of the reference mirror adjacent to the sensor, and the gain drift under the environmental effects. The delay and gain were subsequently solved by virtue of correlation analysis and traversal. On this basis, Qian et al. [19] proposed to solve the gain and delay using a two-dimensional golden section method in 2018, which is essentially a traversal method. However, this method considerably reduced computation, shortened the duration of vibration compensation, and enhanced the computational efficiency while guaranteeing the same effect of vibration compensation as achieved by the approach taken by Wang et al. In 2022, Yao et al. [28] applied the simplified model of transfer function in the vibration compensation for an atomic gravimeter for the first time, and corrected the interference fringe of the atomic gravimeter. In a quiet laboratory, this approach could make the standard deviation of the cosine fitting residual error of the interference fringe attenuate by 58%. Nevertheless, it has not been further tested in a complex vibration environment.

Inspired by the above studies, we propose a vibration compensation approach in this paper to correct the interference fringe of an atomic gravimeter. Based on EO, it allows estimating the simplified model of the transfer function for a reference mirror. The approach is applicable to an atomic gravimeter in a complex vibration environment. As a novel meta heuristic optimization algorithm put forth by Faramarzi et al. in 2019 [29], the EO algorithm can simulate the concentration of inactive matters that enter, leave, or are generated based on the state of dynamic mass balance in a control volume. Among them, each particle with its concentration acts as a search agent and represents a solution for an optimization problem. In this paper, the transfer function between a Raman reflector and a seismometer in an atomic gravimeter is simplified into a “gain-delay” model. The RMSE in the fitting of an atomic interference fringe after the vibration compensation is taken as the fitness and estimated with the EO algorithm, so as to restore the actual vibration of the Raman reflector. Vibration compensation is adopted to minimize the effect of vibration on the gravity measurement and improve the accuracy of the gravity measurement.

This paper is structured as follows: The second part describes the principles of an atomic gravimeter, the basic principles of the simplified model of transfer function, and the method of vibration compensation through the estimation of transfer function. The test environment and equipment and vibration compensation process are detailed in the third part. The fourth part presents the test with a single vibration compensation and 50 vibration compensations, and compares vibration compensation with a genetic algorithm and particle swarm optimization. The test results are listed and analyzed. The fifth part gives the conclusions and lays out the future optimization of vibration compensation.

## 2. Principles and Methods

### 2.1. Vibration Compensation for an Atomic Gravimeter

During the atomic interference, a three-dimensional magneto-optical trap can cool and trap the ^85^Rb atoms emitted from an alkali metal source to form atomic groups. Through polarization gradient cooling, atoms are cooled to the µK level, and then prepared to the state F = 2 in the D2 line. All light fields are turned off for the free fall of atomic groups under the effect of acceleration. Subsequently, phase-locked Raman laser pulses are used to achieve the splitting, reflection, and combination of ^85^Rb atoms at the ground state F = 2 and mF = 0. A Mach–Zehnder interference loop is therefore generated for atoms to transfer the information of gravity into an atomic interference fringe. In the end, the time-of-flight method is employed to detect the fluorescence signal of atoms in the detection area. Thus, the normalized atom population *P* at the state F = 3 and mF = 0 is determined as follows:

(1) $$P=P_{0}+C\cos\phi$$

where *P*_0_ is the DC offset of a fringe; *C* is the contrast of an atomic interference fringe; and *ϕ* is the atomic interference phase from the accumulated paths of atoms during interference, which can be expressed as follows:

(2) $$\phi =(k_{eff}g-2\pi \alpha )T^{2}+\Delta \phi_{vib}$$

where Δ*ϕ_vib_* is the interference phase generated by vibration-induced noise, that is, a term in need of vibration compensation; *a* is the chirp rate of Raman lights; *k_eff_* is the equivalent wave vector of Raman lights; and *T* represents the time interval between two adjacent Raman light pulses. With Formulas (1) and (2), the gravitational acceleration *g* can be determined.

In the above simply outlined operating principles of an atomic gravimeter, Δ*ϕ_vib_* reflects at the phase of the influence of the vibration-induced noise on the atomic gravimeter [30]. Vibration-induced noise is one of the major factors inhibiting the measurement sensitivity of the atomic gravimeter. Particularly, it exerts a significant effect on a Raman reflector, since the vertical vibration of the reflector may straightly introduce noise at the interference phase. For this reason, a seismometer must be mounted adjacent to the reflector to accurately measure its vibration signals. However, vibration may cause the reflector to move. Assuming that the displacement triggered by the vibration in the vertical direction is *d_ref_*(*t*), and its velocity is *v_ref_*(*t*), the Raman light phase change resulted from vibration *φ_ref_*(*t*) is as follows:

(3) $$\varphi_{vib}\left(t\right)=k_{eff}d_{ref}\left(t\right)=k_{eff}\int_{0}^{t}v_{ref}(t^{\prime })dt^{\prime }$$

Within an interference period, the phase shift noise Δ*ϕ* of an atomic gravimeter arising from the phase change in Raman lights is as follows [31]:

(4) $$\Delta \phi =\int_{0}^{2T}s(t)\cdot d\varphi \left(t\right)=\int_{0}^{2T}s(t)\cdot \frac{d\varphi \left(t\right)}{dt}dt$$

where *s*(*t*) is the sensitivity function of an atomic gravimeter with π/2−π−π/2 Raman pulse, and written as the following:

(5) $$s(t)=\{\begin{array}{ll} 0 & t<-T\\ \sin\Omega (T+t) & -T\leq t<-T+\tau \\ 1 & -T+\tau \leq t<-\tau \\ -\sin\Omega t & -\tau \leq t<\tau \\ -1 & \tau \leq t\leq T-\tau \\ -\sin\Omega (T-t) & T-\tau \leq t<T\\ 0 & T\leq t \end{array}$$

where Ω is the Rabi frequency. With Formulas (3) and (4), the phase Δ*ϕ_vib_* generated by the vibration-induced noise within an interference period can be determined as follows [32]:

(6) $$\Delta \phi_{vib}=k_{eff}\int_{0}^{2T}s(t)\cdot v_{ref}(t)dt$$

**Figure 1:**
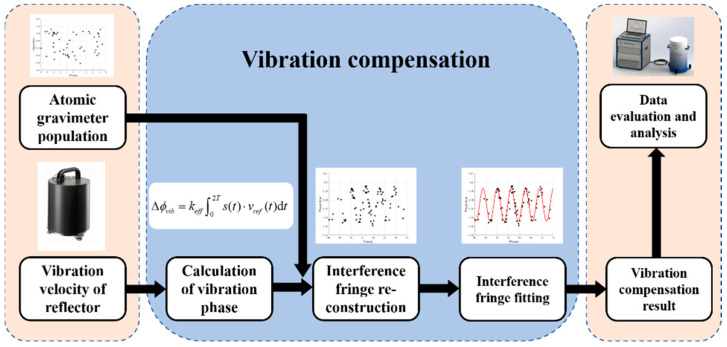
Principles of vibration compensation.

Calculating the phase Δ*ϕ_vib_* is crucial to vibration compensation, while the actual vibration signal *v_ref_*(*t*) of the reflector as given by Formula (6) plays a significant role in this calculation. The principles of vibration compensation are illustrated in Figure 1. The first step is to collect signals. The atomic interference population is detected by the atomic gravimeter. The vertical vibration velocity signal of the Raman reflector is output by the seismometer. After being corrected with the transfer function, the vertical output signal of the seismometer can be regarded as the vibration signal of the Raman reflector. Moreover, the phase shift Δ*ϕ_vib_* caused within the measurement period is calculated through integration. After that, this phase shift can be used for vibration compensation in the original “phase-population” curve generated by the atomic gravimeter. Therefore, a new interference fringe is created to obtain the absolute gravity through fitting.

### 2.2. Simplified Model of Transfer Function

**Figure 2:**
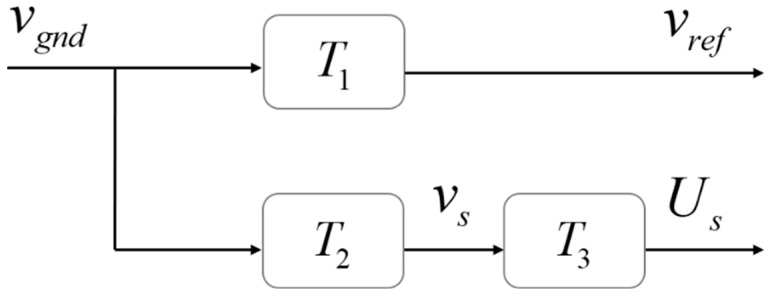
Relationship between a seismometer’s output and a Raman reflector’s vibration.

Presently, vibration-induced noise severely restricts the measurement accuracy of an absolute gravimeter. The measurement of a Raman reflector is significantly affected by the vibration compensation of an atomic gravimeter. In the test, a broadband seismometer was placed under the reflector to measure its vibration signal, and its output was voltage signal *U_s_*, which satisfied the following:

*U*_s_(*t*) = *K*_s_*v*_s_(*t*) (7)

where *K_s_* is the nominal sensitivity of the seismometer, which can be found in its manual, and *v_s_* is the velocity signal that the seismometer is capable of detecting. However, the output signal *U_s_* of the seismometer cannot fully indicate the actual vibration of the reflector *v_ref_* because of its limited performance, mechanical structure, and arrangement. Their relationship is as shown in Figure 2 [18].

In the figure, *T*_1_ is the transfer function from the ground vibration *v_gnd_* to the Raman reflector’s vibration *v_ref_*; *T*_2_ is the transfer function from the ground vibration to the seismometer’s output; and *T*_3_ is the transfer function from the seismometer’s input to its output *U_s_*. As shown in the figure, the transfer function *T* between the actual vibration velocity of the Raman reflector and the voltage signal output by the seismometer satisfies the following formula:

(8) $$T=\frac{U_{s}}{v_{ref}}=\frac{T_{2}\cdot T_{3}}{T_{1}}$$

For this reason, the actual vibration *v_ref_* of the Raman reflector should be restored as accurately as possible using the seismometer’s output *U_s_*, in order to achieve highly accurate compensation. The key to this restoration is to solve the transfer function *T* as correctly as possible, which exerts a direct effect on the vibration compensation. Nevertheless, it is complicated to measure *T*_1_, *T*_2_, and *T*_3_ in the practical condition, so they are reasonably simplified in the existing studies. Analogous to the optical gravimeter [33], a simplified model formed by gain coefficient *K* and delay coefficient *τ* is adopted to estimate the transfer function *T*. This simplified model, also known as “gain-delay” model, is expressed as the following:

(9) $$v_{ref}(t)=Kv_{s}(t+\tau )=\frac{1}{K_{s}}KU_{s}(t+\tau )$$

With the given gain coefficient *K* and delay coefficient *τ*, the phase change caused by the Raman reflector’s vibration is calculated as follows:

(10) $$\Delta \phi_{vib}=k_{eff}\frac{1}{K_{s}}\int_{0}^{2T}s(t)\cdot KU_{s}(t+\tau )dt$$

Theoretically, when the gain coefficient *K* and delay coefficient *τ* are closer to their actual values, the calculated vibration compensation phase Δ*ϕ_vib_* is closer to its actual value. This will lead to better correction of interference fringe and higher accuracy of vibration compensation for an atomic gravimeter.

### 2.3. Equilibrium Optimizer Algorithm

According to the principles of atomic interference gravity measurement, there is a trigonometric function between atom population *P* and interference phase *ϕ*. Therefore, the parameters of the transfer function can be determined by evaluating the fitting of the restructured interference fringe with the cosine wave. Within the atomic interference period, chirp rate is scanned, while atom population and its interference phase are obtained. After deducting the vibration phase Δ*ϕ_vib_* by Formula (3), the “interference phase-population” curve generated after vibration compensation is treated by least square fitting to obtain the cosine fringe. The RMSE of the fringe in the fitting is calculated to find the gain coefficient *K*_opt_ and delay coefficient *τ*_opt_ at the lowest RMSE. The two coefficients are the optimal parameters for the simplified model of transfer function. This is essentially a problem of seeking optimal parameters. For this reason, the EO algorithm can be adopted to determine the optimal parameters for the transfer function. The RMSE at the time of interference fringe fitting after each vibration compensation is selected as the target of optimization, that is, the fitness of this algorithm.

In this test, the EO algorithm was used to search for the optimal parameters for the simplified model of transfer function. Its principles are detailed as follows:

1. Initialize the concentration. Similar to most meta heuristic optimization algorithms, EO algorithm has the concentration initialized by the following:
  (11) $$C_{i}^{init}=C_{max}+rand_{i}\left(C_{max}-C_{min}\right)\;i=1,2,\cdots ,n$$
  where $C_{i}^{init}$ is the initial concentration of the *i*th individual; *C_min_* and *C_max_* represent the minimum and maximum concentration of the individual, respectively; *rand_i_* is a random vector in [0, 1]; and *n* indicates the number of individuals in the population.
2. Build an equilibrium pool and set the candidate solution. The EO algorithm converges to the final state, that is, concentration equilibrium, which is deemed as the optimal state. An equilibrium pool provides a candidate solution for the entire process of optimization, so as to enhance the capability of global optimization. The equilibrium pool is formed by four individuals that have relatively optimal fitness obtained from initialization or iteration, and their mean value is as follows:
  (12) $${\vec{C}}_{eq.pool}=\left\{{\vec{C}}_{eq(1)\;},{\vec{C}}_{eq(2)\;},{\vec{C}}_{eq(3)\;},{\vec{C}}_{eq(4)\;},{\vec{C}}_{eq(ave)}\right\}$$
  where,
  (13) $${\vec{C}}_{eq(ave)}=\sum_{i=1}^{4}{\vec{C}}_{eq(i)}/4$$
  The five individuals in the equilibrium have the same probability of being selected as the solution guiding the optimization. The probability is 0.2.
3. Update the concentration. After constructing the equilibrium pool, the EO algorithm mainly follows the direction of the exponential term $\vec{F}$ in the transition from exploitation to exploration. This exponential term plays a significant role in the update of the algorithm, and can be expressed as the following:

(14) $$\vec{F}=e^{-\vec{\lambda }\left(t-t_{0}\right)}$$

(15) $$t={\left(1-\frac{\;Iter}{Max\_iter}\right)}^{a_{2}\frac{\;Iter\;}{\;Max\_iter\;}}$$

(16) $${\vec{t}}_{0}=\frac{1}{\vec{\lambda }}\ln\left(-a_{1}\cdot sign(\vec{r}-0.5)\left[1-e^{-\vec{\lambda }t}\right]\right)+t$$

where $\vec{\lambda }$ is a random vector in [0, 1]; *Iter* and *Max_iter* are the current number of iterations and the maximum number of iterations; *a*_1_ is a constant, which is normally 2; *sign* is a mathematical symbol function; and $\vec{r}$ is a random number in [0, 1]. By combining Formulas (14) and (16), we can obtain the following:

(17) $$\vec{F}=a_{1}\cdot sign(\vec{r}-0.5)\left[e^{-\vec{\lambda }t}-1\right]$$

Furthermore, a generation probability $\vec{G}$ is introduced into the EO algorithm to improve the accuracy of feasible solution, which further enhances the exploitation capability of the algorithm. The algorithm is then expressed as the following first-order exponential decay:

(18) $$\vec{G}={\vec{G}}_{0}e^{-\vec{\lambda }\left(t-t_{0}\right)}={\vec{G}}_{0}\vec{F}$$

(19) $${\vec{G}}_{0}=\vec{GCP}\left({\vec{C}}_{eq}-\vec{\lambda }\vec{C}\right)$$

(20) $$\vec{GCP}=\{\begin{array}{ll} 0.5r_{1} & r_{2}\geq GP\\ 0 & r_{2}<GP \end{array}$$

where *r*_1_ and *r*_2_ are both random numbers in [0, 1]; *GP* is the generation probability, which is normally 0.5. Above all, the individual concentration iteration update formula of the EO algorithm is as follows:

(21) $$\vec{C}={\vec{C}}_{eq\;}+\left(\vec{C}-{\vec{C}}_{eq}\right)\cdot \vec{F}+\frac{\vec{G}}{\vec{\lambda }V}(1-\vec{F})$$

where *V* is normally 1. In Formula (21), the first term is the concentration in the equilibrium state. The second and third terms represent the variation of concentration, which contributes to the search capability of the algorithm, and triggers the drastic change in individuals close to the local equilibrium state. The third term makes contribution to the exploitation capability, so that more accurate solution can be found within a small concentration range. In addition, the EO has an individual memory-saving mechanism. The fitness of each individual after the *Iter*th (*Iter* ≥ 2) iteration will be compared with the fitness obtained after the (*Iter* − 1)th iteration. If the former is better, the individual’s position and fitness will be updated correspondingly. If not, they will not be updated. The latter will remain unchanged till the next iteration.

## 3. Test

### 3.1. Environment and Equipment

**Figure 3:**
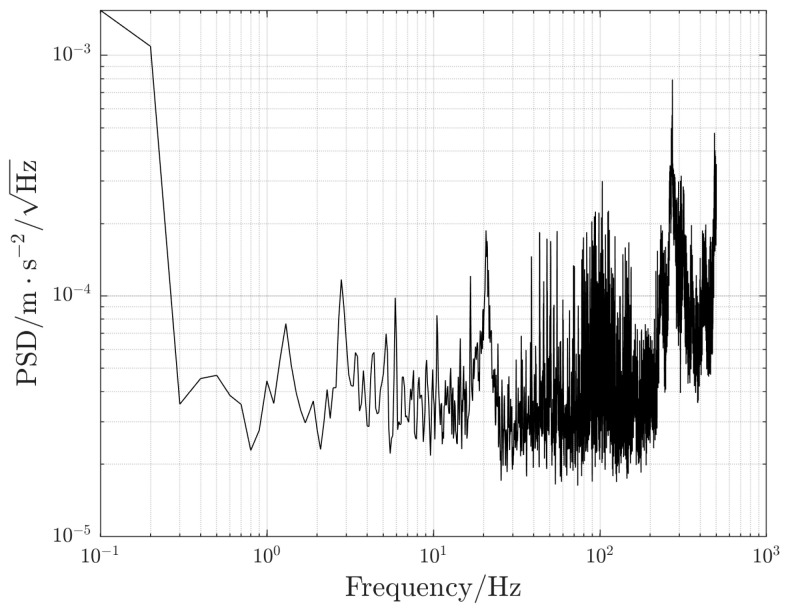
Vibration acceleration power spectrum density at the test place.

**Figure 4:**
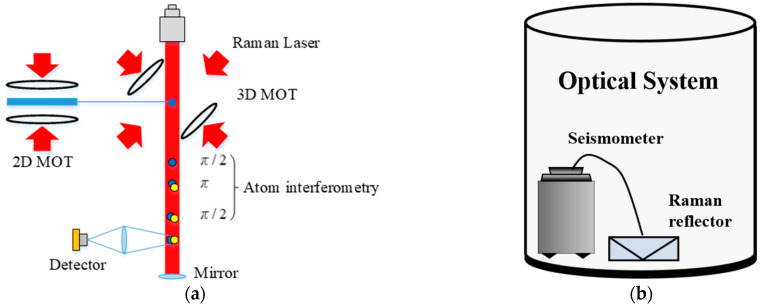
Atomic gravimeter’s optical system. (**a**) Atomic interference with the reflector located at the bottom to reflect Raman lights. (**b**) Inside the optical system, in which the seismometer was placed in the test to respond to the vibration of the reflector in an accurate and sensitive way.

The vibration compensation verification test was carried out in a laboratory in a crowded downtown. The laboratory was on the fourth floor where people often walked around, causing much vibration-induced noise. The complex vibration environment caused an amplitude increase in vibration-induced noise and broadened the frequency band. During the test, equipment was not placed on a vibration isolation platform. The power spectrum density of the floor vibration acceleration was measured as shown in Figure 3. The vibration-induced noise reached the level $10^{-4}\;m\cdot s^{-2}/\sqrt{\mathrm{Hz}}$. The seismometer (CMG-3VL, Güralp Systems Limited., UK) used to measure the vibration signal was a single-axis (only to measure the vibration signal in the vertical direction), low-noise, and broadband ground vibration sensor. It could measure the vibration within the frequency range of 0.003–50 Hz. Its sensitivity was up to 2050 $V/m\cdot s^{-2}$, and its noise level was as low as $3\times 10^{-10}\;g/\sqrt{\mathrm{Hz}}$. Gravity was measured by a WAG atomic gravimeter developed by the Innovation Academy for Precision Measurement Science and Technology, Chinese Academy of Sciences [34]. Its sensitivity was $230\;\mu \mathrm{Gal}/\sqrt{\mathrm{Hz}}$, and its long-term stability reached 5.5 μGal. In the interference process of the atomic gravimeter’s optical system, as shown in Figure 4a, the laser wavelength of the atomic gravimeter was around 780 nm, and the time interval *T* of the Raman pulses was set to 71 ms. An interference fringe was obtained through the linear scanning of the chirp rate. At the middle of the fringe, linear chirp scanning was carried out for Raman detuning. A complete chirp scanning period consisted of 40 atomic interferences, with the first half for positive chirp scanning and the second half for negative chirp scanning. The position of the Raman reflector and the seismometer in the optical system is presented in Figure 4b. The Raman reflector was placed right beneath the interference loop and was suspended in the same direction as the vertical vibration measured by the seismometer. The seismometer is also embedded into the optical system and attached to the centerline of the platform for adjusting the level at the bottom. It is then linked to the Raman reflector through a customized structure so as to respond to the vibration of the reflector in an accurate and sensitive way.

### 3.2. Basic Procedure

The test was conducted in the following procedure: The data of the vibration sensing system was first collected. During the operation of the atomic gravimeter, the atomic interference population and its triggering signal were collected with the output signal of the seismometer at the same time. In this test, the atomic interference gravity measurement was conducted 50 times with 24 s each. By normalized detection, the atomic interference population was gathered. Among 50 chirp scanning periods, each period generated the data of 12 s positive chirp scanning and 12 s negative chirp scanning, which followed the same principles of vibration compensation. From the data, 50 pieces of positive chirp scanning data were extracted for these periods to verify the proposed vibration compensation approach. During each measurement period, a sampling rate of 50 kHz was used to gather the vibration velocity signals from the seismometer at the corresponding time of gravity measurement. The output of the sensor was the voltage, which was denoted by $U_{s,i}(t),i=1,2,\ldots ,50$.

Subsequently, the data of the seismometer were preliminarily processed. The transfer function of the seismometer was not absolutely equivalent to the simplified “gain-delay” model of the transfer function. The difference between them might make it impossible to accurately restore the actual vibration of the Raman reflector, resulting in insufficient vibration compensation. For this reason, the data of the seismometer were preliminarily processed. Before implementing the vibration compensation algorithm, a digital lead compensation filter was employed to dynamically filter the original data. The transfer function was denoted by *H*(*f*) and expressed as in Formula (22) [35]. It was a recursive infinite impulse response filter with the angular frequency *f*_a_ and *f*_b_, and normally *f*_a_ < *f*_b_.

(22) $$H(f)=\frac{1+jf/f_{a}}{1+jf/f_{b}}$$

By adjusting *f*_a_ and *f*_b_, the filter could be used to compensate for the high-frequency amplitude attenuation of the seismometer’s transfer function and reduce the phase lag. Numerically, it could increase the bandwidth of the seismometer, so that the filtered transfer function of the seismometer was closer to the simplified “gain-delay” model of transfer function. In the test, we took *f*_a_ = 70 Hz and *f*_b_ = 700 Hz.

At last, the vibration compensation algorithm was employed for data post-processing. The collected data were synchronized with the atomic interference population through the triggering signal. The simplified model of the transfer function was then estimated by the EO algorithm. The seismometer output *U_s,i_*(*t*) should restore the actual vibration *v_ref_*(*t*) of the Raman reflector as accurately as possible. The basic process and pseudo-code (see Algorithm 1) of the EO algorithm are as follows:

Step 1: Set the basic parameters and determine the population size and the maximum number of iterations. Set the proportion of gain and the search space of delay, and import the transition probability *P* sequence corresponding to the scanning phase sequence. Import the data of the seismometer output *U_s,i_*(*t*);

Step 2: Initialize the particle population, and set the initial value of four candidate solutions to be infinite;

Step 3: Determine the lowest RMSE of the interference fringe after fitting and vibration compensation for each set of data, and take it as the target function. Calculate the fitness of every individual. Select four individuals in terms of fitness, and calculate their mean value. Construct an equilibrium pool;

Step 4: Save the memory of these individuals and conserve the best individual of the current population. Update the exponential term and the generation rate;

Step 5: Select an individual randomly by equal probability from the constructed equilibrium pool and update the individual’s concentration by Formula (21);

Step 6: Move to Step 7 if the algorithm satisfies the condition for termination, that is, if the set maximum number of iterations is reached. Move to Step 3 to repeat the next search if not;

Step 7: Output the optimal individual of the equilibrium pool, that is, the optimal solution with the delay coefficient *τ*_opt_ and gain coefficient *K*_opt_ to calculate the corrected interference fringe and its RMSE at the time of fitting.
**Algorithm 1: Pseudo-Code of the EO algorithm**1: Set parameters and import data 2: Initialize particle concentration 3: While Iter<Max_iter
4: For 5:  Calculate the fitness of all particles, and select the optimal particle6: End for7: Construct an equilibrium pool with Formula (12) and (13) 8: For
k=1:N
9:  Select the candidate solution randomly from the equilibrium pool10.  Generate $\vec{\lambda }$ and $\vec{r}$ randomly11:  Calculate the exponential term $\vec{F}$ with Formula (14) to (17)12:  Calculate the generation rate $\vec{G}$ with Formula (18) to (20)13:  Update the concentration $\vec{C}$ with Formula (21) 14: End for15: t=t+1
16: End while

## 4. Results and Analysis

**Figure 5:**
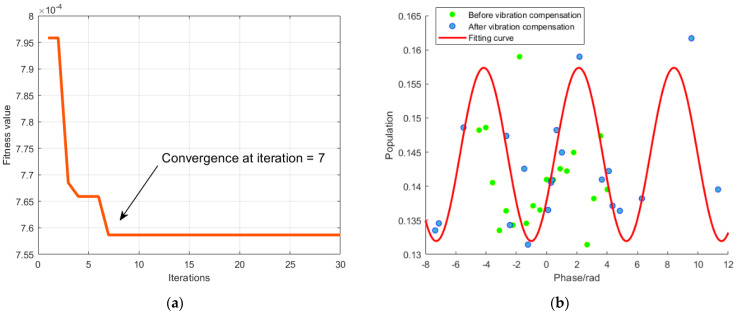
Single vibration compensation with the EO algorithm. (**a**) Fitness after seeking the optimal parameters of transfer function. (**b**) Atomic interference fringe and fitting curve before and after vibration compensation.

The software MATLAB designed by a U.S. company MathWorks was used to verify the effectiveness of the EO algorithm. The particle population was set to 50, and the maximum number of search iterations was 30. The proportion of gain and the search space of delay were K∈[0.5,2.5] and *τ* ∈ [−1,6], respectively. We collected a set of test data first. The set of data contained the atomic interference population collected by normalized detection from the atomic gravimeter detection system and the output voltage of the seismometer. Then, the parameters were estimated for the simplified model of transfer function using the EO algorithm. Vibration compensation was made to obtain the iterative optimization curve and the atomic gravimeter interference fringe curve. As shown in Figure 5a, the fitness hit the bottom and converged after the seventh iteration. The local optimal gain coefficient *K*_opt_ was determined to be 1.9087, and the optimal delay coefficient *τ*_opt_ was 4.8765 ms. Figure 5b showed the atomic interference population before (in green) and after (in blue) the vibration compensation. Evidently, the population before compensation was confusing and disordered. After compensation at the vibration phase, the population tended to be a cosine function. The vibration compensation algorithm still achieved a good correction of the interference fringe in a complex vibration environment. It could be approximately fit as a cosine curve (in red). Certainly, there were still some small deviations between the population and the fitting curve because of other external factors such as the seismometer’s limited performance and the atomic gravimeter being disturbed by other noise. Consequently, the measured atomic interference fringe did not form a perfect cosine curve.

After that, ten sets of data were further collected. The same parameters for the simplified model of transfer function were estimated for each set of data using the EO algorithm. The results are given in Table 1, including the optimal gain coefficient *K*_opt_ and the optimal delay coefficient *τ*_opt_. For the ten sets of data, the calculated values were *K*_opt_ 1.9251 ± 0.2450 and *τ*_opt_ 3.7864 ± 0.7116 ms. By comparison, the RMSE of the corrected fringe after compensation was significantly lower than the RMSE from the fitting with the original interference fringe before the vibration compensation. Moreover, the RMSE attenuation ratio *σ* was also calculated. The mean attenuation reached 37.53%, and the max one was even up to 48.97%. As revealed by the measurement results with single vibration compensation (one set of data) and ten sets of data, it was feasible and effective to estimate the parameters for the simplified model of transfer function using the EO algorithm. This could satisfyingly restore the actual vibration of the Raman reflector. Additionally, the vibration compensation algorithm actualized the correction and fitting of the original interference fringe, and the RMSE at the time of fitting attenuated dramatically after compensation.

**Figure 6:**
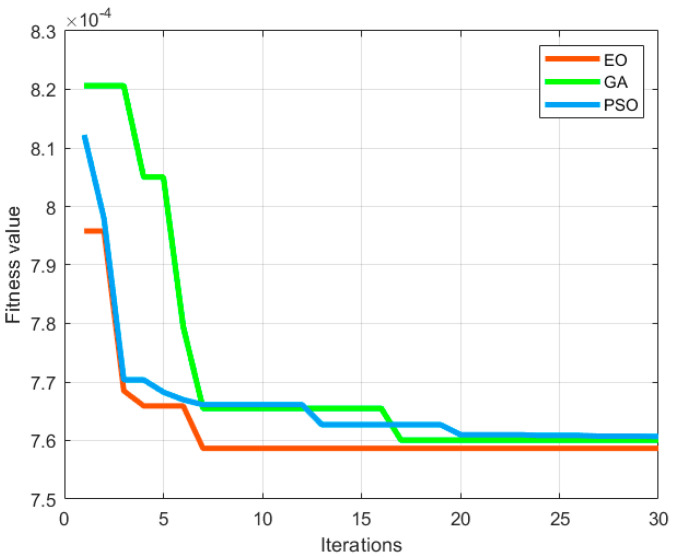
Comparison of fitness curves from the optimization of parameters for transfer function with single vibration compensation.

Two classic optimization approaches were adopted for iterative optimization, that is, the genetic algorithm (GA) and particle swarm optimization (PSO), and then they were compared with the EO algorithm. The performance evaluation indicators were first determined. As for the single vibration compensation, the iterative optimization curve was analyzed to assess the convergence rate of the algorithms, while the minimum fitness and RMSE attenuation ratio were calculated to evaluate the search capability of the algorithms. The duration of the optimization search was calculated to determine the optimization speed of the algorithms. The residual error Δ*g* of gravity measurement was employed to evaluate the uncertainty of gravity measurement. The same population and maximum number of iterations were used for the three algorithms. The algorithms were compared with single vibration compensation for the same set of data. The iterative optimization curve is given in Figure 6. As shown in the figure, the EO algorithm, the GA algorithm, and the PSO algorithm converged at the 7th, 17th, and 20th iterations, respectively, and their minimum fitness was 7.5866 × 10^−4^, 7.6006 × 10^−4^, and 7.6074 × 10^−4^, respectively. It is evident that the EO algorithm converges faster than the other two algorithms in the optimization of parameters for the simplified model of the transfer function. Hence, the EO algorithm can achieve the lowest fitness and the best optimization.

Additionally, other performance indicators were compared for the single vibration compensation, as given in Table 2. For the EO, GA, and PSO algorithms, the optimization search durations were 19.35 s, 46.32 s, and 56.23 s, respectively, and the RMSE attenuation ratios of the fringe fitting were 30.18%, 26.27%, and 27.99%, respectively. Compared with the GA and PSO algorithms, the EO algorithm performed better in search, and realized a higher RMSE attenuation ratio in fringe fitting, so that it could make better compensation for the influence of the vibration phase on the atomic gravimeter. After estimation, the simplified model of the transfer function received vibration compensation. After the atomic interference fringe fitting, the residual error Δ*g* of the gravity measurement, which is different from the actual gravity by a gravity standard *g*_0_, was calculated. The effect of vibration compensation was added to the gravity measurement. Theoretically, vibration-induced noise is one of the major restrictions to the measurement accuracy of an atomic gravimeter in a complex vibration environment. Therefore, vibration compensation exerts a direct effect on the gravity measurement of the atomic gravimeter. In this test, the residual errors Δ*g* of the gravity measurement with the EO, GA, and PSO algorithms were 444.25, 812.35 μGal, and 864.72 μGal, respectively, which proved such a direct effect. In general, the EO algorithm can better search the parameters for the simplified model of the transfer function and restore the vibration signal. Through the compensation at the vibration phase, it delivers a better gravity measurement.

**Figure 7:**
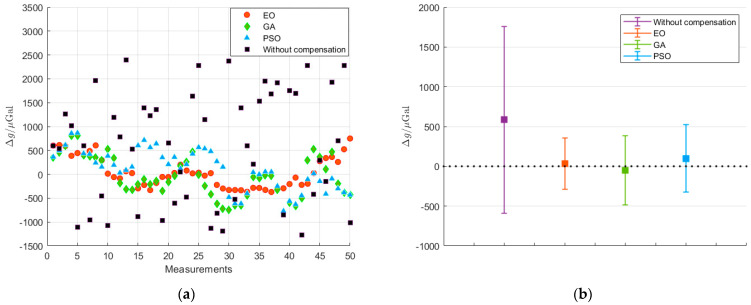
Measurement results after 50 vibration compensations. (**a**) Δg; (**b**) mean value with error band.

In order to further verify the performance of the algorithms, the test was repeated 50 times to gather the results of vibration compensation. We adopted several indicators to evaluate the effect of vibration compensation for the algorithms, including the mean value, standard deviation, and inhibitive factor γ of the residual error Δ*g* of gravity measurement. Among them, γ was defined as the ratio of the gravity measurement resolution, before and after compensation. Evidently, the greater inhibitive factor demonstrated a better contribution of vibration compensation to the improvement of the gravity measurement resolution. Figure 7a gives the measured Δ*g* for the EO, GA, and PSO algorithms after 50 vibration compensations to eliminate the effect of vibration on the gravity measurement of the atomic gravimeter. The mean value of Δ*g* with an error band is shown in Figure 7b. The calculation results are presented in Table 3. The mean values of Δ*g* for the EO, GA, and PSO algorithms are 33.96 µGal, 52.52 µGal and 99.94 µGal, respectively, and their standard deviations are 323.53 µGal, 433.93 µGal, and 424.67 µGal, respectively. Evidently, the mean value and standard deviation of Δ*g* are significantly optimized after vibration compensation. Among these algorithms, the EO algorithm shows the best optimization and delivers the best gravity measurement.

**Figure 8:**
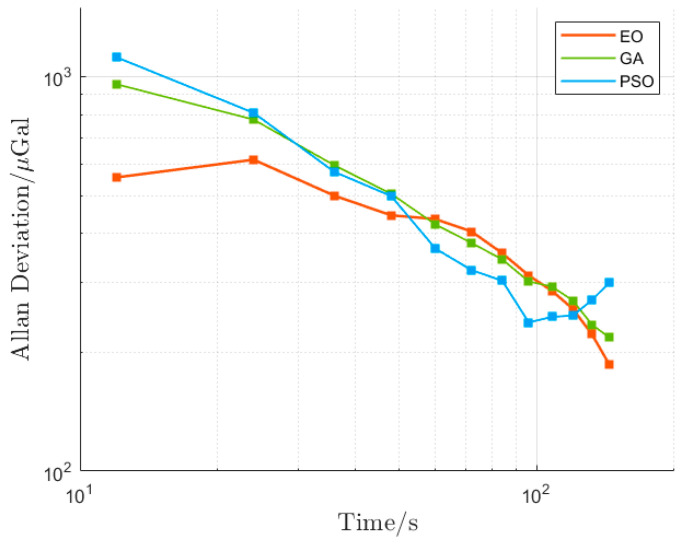
Allan deviation of gravity measurement.

In a precision measurement, the Allan deviation can be used to assess the resolution and stability of an atomic gravimeter. In order to further evaluate and analyze the influence of the vibration compensation algorithms on gravity measurement, the Allan deviation of Δ*g* was calculated (without considering the solid earth tide model since gravity measurement was conducted within a short period). The results, as shown in Figure 8, visually demonstrate that the Allan deviation curves of the EO, GA, and PSO algorithms are not significantly different from each other. The calculated resolution and inhibitive factor γ are given in Table 3. The gravity measurement resolutions of the EO, GA, and PSO algorithms are 186.70 μGal@144s, 218.30 μGal@144s, and 300.20 μGal@144s, respectively. Additionally, γ is 6.77, 5.79, and 4.21, respectively. Evidently, the EO algorithm is still superior to the other two algorithms.

## 5. Conclusions

The calculation of the vibration phase Δ*ϕ_vib_* is crucial to the vibration compensation of an atomic gravimeter. It is essentially the restoration of an actual vibration of the Raman reflector. When the estimation of the transfer function is more accurate, the calculated Δ*ϕ_vib_* is closer to its real value. This will ensure a better correction of the interference fringe for the atomic gravimeter, and greater accuracy in vibration compensation. In this paper, a vibration compensation approach is proposed with a simplified model of the transfer function estimated for the Raman reflector with the EO algorithm and is used to correct the interference fringe of an atomic gravimeter in a complex environment. In this approach, the practical transfer function between the vibration signal of the Raman reflector and the actual output signal of the seismometer in an atomic gravimeter is simplified into a “gain-delay” model. After analyzing the interference fringe detected by the gravimeter and the output of the seismometer, the RMSE in the fitting of the atomic interference fringe after compensation is taken as the fitness to estimate the above transfer function. In this way, the actual vibration signal of the Raman reflector can be restored in a more accurate way. This approach can adaptively estimate the most suitable transfer function for different measurement environments to achieve the best correction of an atomic interference fringe. Then, the optimal gravity measurement will be realized through vibration compensation. As proved by the test results, the proposed approach to estimating the parameters for the simplified model of transfer function is feasible and effective in a complex vibration environment. It could restore the actual vibration of the Raman reflector satisfactorily. The original interference fringe was corrected and fitted by virtue of the vibration compensation. The RMSE at the time was noticeably attenuated after compensation. Its mean value reached 37.53% and even went up to 48.97%. After being added to the gravity measurement through vibration compensation, the obtained residual error of gravity measurement was 33.96 ± 323.53 µGal. Compared with the other algorithms for parameter optimization, the proposed approach realizes faster convergence, lower fitness, and better optimization. Moreover, it can deliver a more accurate gravity measurement with a lower error.

Nevertheless, the test results are not as good as expected, which leaves room for further improvement of the proposed vibration compensation approach. Apart from further optimizing the algorithm for the estimation of the simplified model of transfer function, improvements can be made in the following aspects:

- Optimization of a sensor for vibration measurement. The narrow bandwidth of a sensor leads to severe distortion of measured vibration, making it impossible to restore the vibration of a Raman reflector fully and truly. For this reason, a sensor with a larger bandwidth is often selected. Furthermore, the self-noise of the sensor should be taken into account, since it involves the internal electronic noise and the drift triggered by the change in the external environment. Several sensors of the same type can be combined to test their self-noise.
- Proportion of noise at other phases. Vibration-induced noise is one of the major restrictions to the measurement sensitivity of an atomic gravimeter, but noise also exists at other residual phases and should not be ignored. When the noise at other phases takes a larger proportion of the total phase noise of the atomic gravimeter, it is necessary to further explore its influence on the vibration compensation algorithm.
- Test on mobile platforms and in more complex vibration-induced noise environments. In a highly noisy environment, the phase noise caused by ground motion may spread over multiple atomic interference fringes, making it more difficult to implement vibration compensation.

## Figures and Tables

**Table 1:** Measurement results with ten sets of data.

	Parameter		RMSE		
	$K_{\mathrm{opt}}$	$\tau_{\mathrm{opt}}$	Before Compensation/10^−3^	After Compensation/10^−3^	σ
1	1.9087	4.8765	9.5675	6.6804	30.18
2	2.1258	3.7460	8.4996	5.7656	32.17
3	1.9124	4.3657	7.8627	5.7318	27.10
4	2.1182	3.3427	6.4535	4.5149	30.04
5	1.6203	4.0268	7.8033	4.1538	46.77
6	1.5682	2.0576	8.7150	5.4374	37.61
7	2.0729	4.2040	9.6066	6.3619	33.78
8	1.7019	3.7127	10.9827	6.3976	41.75
9	2.3949	4.0057	6.5932	3.3646	48.97
10	1.8281	3.5265	9.7725	5.1822	46.97
Average	1.9251	3.7864	-	-	37.53
Standard Deviation	0.2450	0.7116	-	-	-

**Table 2:** Comparison of the algorithms with single vibration compensation.

	EO	GA	PSO
Time of searching the optimization/s	19.35	46.32	56.23
RMSE attenuation ratio of fringe fitting/%	30.18	26.27	27.99
Δ*g*/μGal	444.25	812.35	864.72

**Table 3:** Comparison of results from 50 vibration compensations.

		EO	GA	PSO
Average of Δ*g*/μGal	before compensation	586.53		
	after compensation	33.96	−52.52	99.94
Standard deviation of Δ*g*/μGal	before compensation	1174.93		
	after compensation	323.53	433.93	424.67
Resolution/μGal@144s	before compensation	1263.30		
	after compensation	186.70	218.30	300.20
γ		6.77	5.79	4.21

## Data Availability

Data underlying the results presented in this paper are not publicly available at this time but may be obtained from the authors upon reasonable request.
